# Supplementary figures and images for: Ligand-coupled conformational changes in a cyclic nucleotide-gated ion channel revealed by time-resolved transition metal ion FRET
Source: eLife. 2024 Dec 10;13:RP99854. doi: 10.7554/eLife.99854 (PMC11630820; doi:10.7554/eLife.99854)

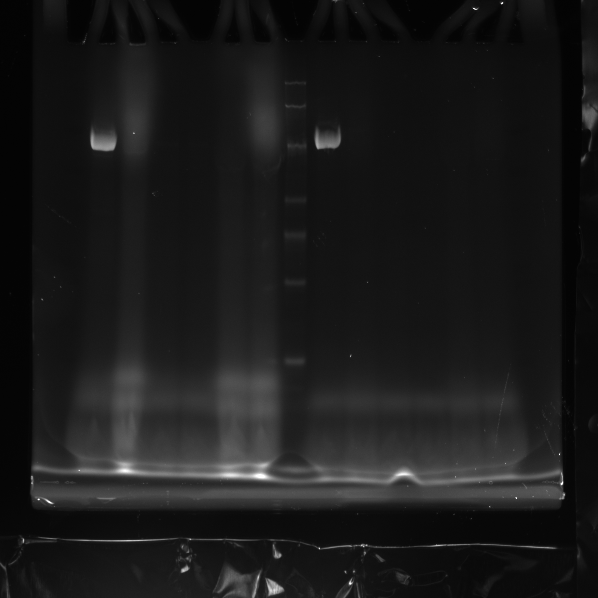

Supplement: Figure 2—source data 1. [file elife-99854-fig2-data1.zip › Figure 2-Source Data 1.tif]
